# Lupus catatonia in a young girl who presented with fever and altered sensorium

**Published:** 2014

**Authors:** Alia Ali, Azeem Taj, Misbah -uz-Zehra

**Affiliations:** 1 Alia Ali, FCPS, Senior Registrar, Department of Medicine, Postgraduate Medical Institute, Lahore, Pakistan.; 2 Azeem Taj, FCPS, Associate Professor, Department of Medicine, Postgraduate Medical Institute, Lahore, Pakistan.; 3 Misbah-uz-Zehra, Trainee Registrar, Department of Medicine, Postgraduate Medical Institute, Lahore, Pakistan.

**Keywords:** Catatonia, SLE

## Abstract

We report a case of 20 Years old girl who presented with catatonia resulting from cerebral lupus. There are few cases of catatonia being described in Systemic Lupus Erythmatoses (SLE). The patient presented to us with fever and altered sensorium. She was initially treated on lines of Acute Bacterial Meningitis/encephalitis but lumbar puncture examination and CT scan showed no evidence of these conditions. Patient’s behavior was also not improved after this treatment and she further deteriorated in the sense that she exhibited mutism, negativism and psychosocial withdrawal. Psychiatric analysis was done and she was found to be having catatonia and on further investigation came out to be a case of SLE. Keeping in mind her previous history of joint pains, oral ulcers and alopecia her autoimmune profile such as ANA and dsDNA was done that came out to be positive. Patient responded to treatment with steroids, Hyroxychloroquine and azathioprine in addition to clonazepam and fluoxetine for her catatonic behavior. Thus this case history illustrates the importance of considering organic disease in patients presenting with catatonia.

## INTRODUCTION

Catatonia is a clinical syndrome characterized by psychosocial withdrawal, negativism, mutism, and posturing together with signs of rigidity and waxy flexibility. There may also be periods of excitement or bizarre repetitious behavior. Catatonia is frequently assumed to be diagnostic of Schizophrenia, although it is known to occur rarely in a number of metabolic and structural diseases of the brain.^1^

Systemic Lupus Erythmatoses (SLE) is a chronic autoimmune disease characterized by multisystem involvement. Neuropsychiatric disturbances ranging from cognitive dysfunction, neuropathy, headache, convulsion, psychosis, mood disorders, and delirium to life threatening coma were found in 50-70% of SLE Patients.^2^^,^^3^ There are few cases of catatonia described in SLE.^4^^-^^6^ We report a case of catatonia resulting from cerebral involvement by Systemic lupus erythmatosus (SLE), in which investigations confirmed the organic nature of the disease.

## CASE REPORT

A 20 year old girl presented with one week history of high grade fever and altered sensorium. Systemic inquiry revealed psychosocial withdrawal, decreased oral intake, minimal speech and inability to move for last one week. There were also complaints of urinary and fecal retention for the same duration. She had also been complaining of polyarthralgias, oral ulcers and alopecia for last one year. No medical attention was sought for these complaints. She was also diagnosed as having hypertension seven months ago associated with headache and blurring of vision and was taking tablet Losartan 25mg twice daily. In past she had a history of pulmonary tuberculosis twice, first at the age of 6 years then again at the age of 11 years. She took Anti tuberculous therapy for nine months and then for whole year.

**Fig. 1:**
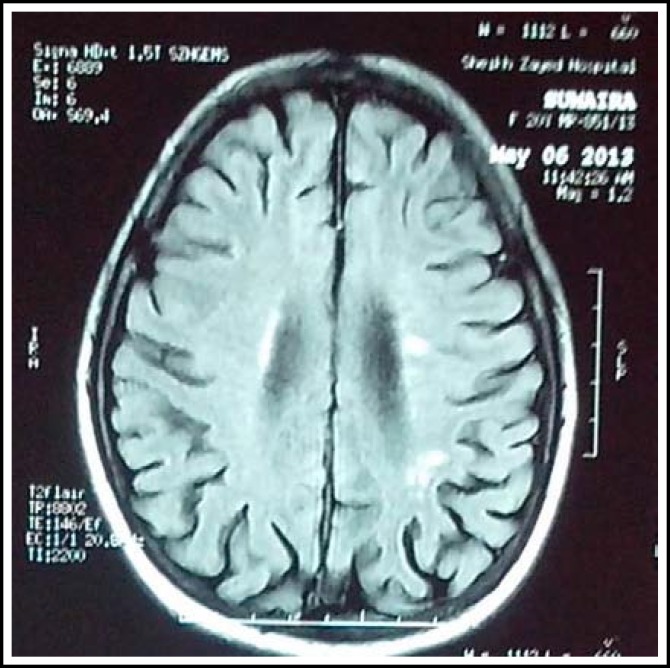
MRI showing demyelination in deep white matter (arrow).

On presentation she was febrile with temperature of 100^o^F, B.P was 150/110mmHg. Pulse was 130 beats/min and regular. She was conscious but withdrawn and vacant. She was completely mute and did not obey commands. Periodically she would move her eyes conjugately as if inspecting her surroundings, but would only occasionally and briefly fixate on an object or her attendants. She made no other spontaneous movement, but would stand if lifted out of bed and would take a few steps if led. The tone in all limbs was variably increased and she demonstrated waxy flexibility, maintaining indefinitely any posture into which her limbs were placed. Planters were both down going with normal reflexes. Although her neck was rigid in all directions but kerning sign was negative. The remainder of neurological examination was normal. No abnormalities were found in the cardiovascular, respiratory or abdominal systems.

Her labs revealed Hemoglobin of 11.7g/dl, WBC count of 12x10^ 3^/uL and platelet count of 235x10^3^/uL. ESR was 76mm in first hour. Serum creatinine was 1.6 mg/dL and 24 hr urinary proteins were raised to 720mg. CSF examination was normal. Serum electrolytes, liver function test, serum calcium, phosphate and uric acid were within normal limits. Chest radiograph showed patchy consolidation and calcified hilar lymph nodes consistent with past tuberculosis. CT scan brain was also normal. Initially she was treated with antipyretics, high dose ceftriaxone as well as acyclovir on the suspicion of acute bacterial meningitis/encephalitis which was discontinued later on when her culture reports (blood, urine, CSF and sputum) did not reveal any growth. Her clinical condition did not improve so MRI brain and expert psychiatric evaluation were planned.

MRI brain showed central white matter nodular demyelination most likely post inflammatory (Fig.1). On the basis of MRI brain and her previous history of joint pains her autoimmune profile was planned. Her antinuclear antibody came out to be strongly positive and homogenous. Anti dsDNA was also markedly elevated with a value of 95.3%. Complement levels were suppressed. So I/V methylprednisolone was started 500mg/d.

Meanwhile psychiatric evaluation confirmed the presence of depressive catatonia and she was started on clonazepam and fluoxetine in escalating doses. Within 3 days the catatonic features diminished and she began to respond to simple commands and after a week she also started talking. In addition to above mentioned treatment she was also given Hydroxy-choloroquin 200mg/day and Azathioprine initially 50mg/day then 100mg/day and discharged with follow-up after one month. On follow up she was able to move around on her own, can make small conversations, oral intake improved and symptoms of urinary and fecal retention were settled. She is still in phase of recovery and on regular follow up.

## DISCUSSION

Catatonia is a syndrome of physical and behavioral abnormalities that can result from psychiatric, neurological or medical illness. Although systemic lupus erythmatosus (SLE) is commonly known to cause neurological and psychiatric manifestations it has only rarely been reported to cause the catatonic syndrome.^4^^-^^6^ The association of catatonic symptoms in SLE demonstrated that catatonic disorder due to general medical conditions should be considered in every patient with catatonic signs.^1^

 In nearly all previously reported cases, except few^7^, the diagnosis of catatonia was reported in patients with an established diagnosis of lupus. We report a case in which a young girl, not a diagnosed case of SLE, but having previous history of joint pains, oral ulcers and alopecia now presented with catatonia that partially responded to standard treatment with benzodiazepines, suffered a long and complicated hospital course, and was eventually diagnosed with lupus. With initiation of treatment for lupus her symptoms of catatonia remitted. Pooled case reports suggest that catatonia due to an underlying general medical condition and catatonia due to a psychiatric illness can be treated similarly and that the catatonic symptoms and the underlying illness must be addressed in both types.^8^

The brain is commonly involved in SLE, and she had obvious organic central nervous system disease. The MRI brain appearances were compatible with cerebral lupus. Cerebral lupus may occur without signs of disease activity in other organs, and psychotic behavior may precede the development of other features of the condition by months or years.^9^ Although acute meningitis or encephalitis may result in catatonia^10^ this did not appear to be a case in our patient as her lumbar puncture examination and imaging studies were not suggestive of these diagnoses. On the other hand Aseptic meningitis/encephalitis can be a part of neuropsychiatric manifestations of SLE but as her investigations did not support these conditions, it is therefore likely that the catatonic state in our patient was independent manifestation of this condition. Psychiatric disorders are well recognized in SLE, and catatonia has previously been reported as already mentioned.

This case history illustrates the importance of considering medical causes in the diagnosis and treatment of psychiatric disorders, especially the catatonic syndrome. SLE should therefore be included in the differential diagnosis of patients presenting with the catatonic syndrome.^11^
